# Characterization of a novel zebrafish (*Danio rerio*) gene, *wdr81*, associated with cerebellar ataxia, mental retardation and dysequilibrium syndrome (CAMRQ)

**DOI:** 10.1186/s12868-015-0229-4

**Published:** 2015-12-23

**Authors:** Fusun Doldur-Balli, Mehmet Neset Ozel, Suleyman Gulsuner, Ayse B. Tekinay, Tayfun Ozcelik, Ozlen Konu, Michelle M. Adams

**Affiliations:** Department of Molecular Biology and Genetics, Bilkent University, Ankara, Turkey; UNAM-Institute of Materials Science and Nanotechnology, Bilkent University, Ankara, Turkey; Interdisciplinary Program in Neuroscience, Bilkent University, Ankara, Turkey; Molecular Biology and Genetics Department Zebrafish Facility, Bilkent University, Ankara, Turkey; Psychology Department, Bilkent University, Ankara, Turkey

**Keywords:** *wdr81*, Zebrafish, RACE, qRT-PCR, In situ hybridization

## Abstract

**Background:**

*WDR81* (WD repeat-containing protein 81) is associated with cerebellar ataxia, mental retardation and disequilibrium syndrome (CAMRQ2, [MIM 610185]). Human and mouse studies suggest that it might be a gene of importance during neurodevelopment. This study aimed at fully characterizing the structure of the *wdr81* transcript, detecting the possible transcript variants and revealing its expression profile in zebrafish, a powerful model organism for studying development and disease.

**Results:**

As expected in human and mouse orthologous proteins, zebrafish wdr81 is predicted to possess a BEACH (Beige and Chediak-Higashi) domain, a major facilitator superfamily domain and WD40-repeats, which indicates a conserved function in these species. We observed that zebrafish *wdr81* encodes one open reading frame while the transcript has one 5′ untranslated region (UTR) and the prediction of the 3′ UTR was mainly confirmed along with a detected insertion site in the embryo and adult brain. This insertion site was also found in testis, heart, liver, eye, tail and muscle, however, there was no amplicon in kidney, intestine and gills, which might be the result of possible alternative polyadenylation processes among tissues. The 5 and 18 hpf were critical timepoints of development regarding *wdr81* expression. Furthermore, the signal of the RNA probe was stronger in the eye and brain at 18 and 48 hpf, then decreased at 72 hpf. Finally, expression of *wdr81* was detected in the adult brain and eye tissues, including but not restricted to photoreceptors of the retina, presumptive Purkinje cells and some neurogenic brains regions.

**Conclusions:**

Taken together these data emphasize the importance of this gene during neurodevelopment and a possible role for neuronal proliferation. Our data provide a basis for further studies to fully understand the function of *wdr81*.

**Electronic supplementary material:**

The online version of this article (doi:10.1186/s12868-015-0229-4) contains supplementary material, which is available to authorized users.

## Background

*WDR81* (WD repeat-containing protein 81) is associated with cerebellar ataxia, mental retardation and disequilibrium syndrome (CAMRQ2, [MIM 610185]) [1, 2], also referred to as Uner Tan syndrome [3]. The missense mutation, *WDR81* p.P856L, which is located in exon 1 of the *WDR81* isoform 1, results in significant decreases in the volume of the cerebellum and corpus callosum [1]. *WDR81* has also been found to be mutated in >10 % of the colorectal cancer cell lines [4]. A mouse study of a mutant line *nur5*, carrying a mutation in the predicted major facilitator superfamily domain of the Wdr81 protein, showed a similar phenotype with CAMRQ [5].

The structure of the WDR81 protein has been predicted and its expression pattern have been described in both humans and mice. The human WDR81 is thought to include a BEACH (Beige and Chediak-Higashi) domain on the N-terminus, a major facilitator superfamily (MFS) domain following BEACH domain, and six WD40 repeats on the C-terminus. The analysis of the protein sequence led to the hypothesis that WDR81 might be a transmembrane protein. The human *WDR81* is ubiquitously expressed and the highest level of expression was detected in the cerebellum and corpus callosum among other human brain regions [1]. In silico domain predictions for the mouse Wdr81 protein also indicated that it is likely to be a transmembrane protein composed of a BEACH, an MFS, and six WD40 repeat domains [5]^.^*Wdr81* expression was found to be increased in Purkinje cells and the molecular layer of cerebellum in the mouse embryonic brain [1]. Wdr81 also has been detected in the neurons of the deep cerebellar nuclei, the brainstem, the photoreceptors and the other retinal layers of adult wild type mice [5].

While the exact function of WDR81 is yet unknown, information about the predicted domains of the protein might indicate its potential function. Proteins containing BEACH domains are suggested to act as scaffold proteins. They are commonly large proteins and are proposed to function in various membrane-related events such as vesicle fusion and fission, and thus would be involved in vesicular trafficking, membrane dynamics, synapse formation, apoptosis, autophagy and receptor signaling [6]. The MFS domain takes place in single-polypeptide secondary carrier proteins, which transport small molecules in response to chemiosmotic ion gradients [7]. WD40 domain proteins function in several cellular processes, mainly signal transduction, cytoskeleton assembly, regulation of transcription, chromatin dynamics, cell cycle control, apoptosis, and vesicular trafficking by providing a platform for protein–protein or protein-DNA interactions [8]. Thus, the WDR81 protein has the potential to alter function in many areas of the central nervous system.

In silico analysis of zebrafish wdr81 resulted in the prediction of the identical conserved domains observed in the human and mouse orthologue proteins. This finding emphasizes a conserved function in these three species. In the present study, we aimed at fully characterizing the structure of the *wdr81* transcript, searching for the presence/absence of possible transcript variants and revealing its temporal and spatial expression in zebrafish. This is the first report of the characterization of the transcript structure and expression of *wdr81* in zebrafish. We performed rapid amplification of cDNA ends (RACE) method to characterize the 5′ and 3′ ends of the cDNA, and amplified the open reading frame (ORF). We examined the expression of the zebrafish orthologue of mammalian *WDR81* (zebrafish *wdr81*) by employing quantitative real-time polymerase chain reaction (qRT-PCR) and in situ hybridization on whole mount embryos, adult brain and eye tissue sections. Our findings on the transcript structure and expression of the gene of interest will give rise to further research in order to fully understand the function of *wdr81* in zebrafish, which is a powerful model organism providing ease of genetic manipulations.

## Methods

### Zebrafish and embryos

Zebrafish (*Danio rerio*) AB strain were used and embryos were obtained from breeding pairs and grown in E3 medium [9]. Both zebrafish and embryos were housed in the zebrafish facility at Bilkent University, Department of Molecular Biology and Genetics, Ankara, Turkey. All fish and embryos were maintained at 28 °C under 14 h light; 10 h dark cycle. The animal protocol for this study was approved by the Bilkent University Local Animal Ethics Committee (HADYEK) with the approval date: October 10, 2010 and no: 2010/31.

### Amplification of the open reading frame of *wdr81*

Thirty 24 hours post fertilization (hpf) embryos and five 10 months old male zebrafish brains were pooled separately and cDNAs were synthesized from total RNA (04379012001, Transcriptor First Strand cDNA Synthesis Kit, Roche, USA). Embryo and brain cDNAs were amplified by using 10 primer pairs, which were designed to span the ORF of zebrafish *wdr81* transcript according to Ensembl (ENSDART00000156621) (Table 1). Negative control samples that did not include reverse transcriptase during cDNA synthesis (−RT) were also included to the experiment. PCR products were run on a 1 % agarose gel.

**Table 1:** Primer sets for amplification of the open reading frame of zebrafish *wdr81*

	Primer sequence	Annealing temperature (°C)	Expected amplicon size (bp)
Pair 1			
F	5′-GCAAACAGTGCAGAGCTTCTT-3′	60	897
R	5′-GCTGCTCATCAACTGCAATATC-3′		
Pair 2			
F	5′-CCTATCCACCTGCTCAGCTC-3′	60	874
R	5′-AACATGGCTGCATAGCACAG-3′		
Pair 3			
F	5′-CCAGATCTTGGTGGACCAGT-3′	60	909
R	5′-TCTGTGAAGCATGGCAGTTC-3′		
Pair 4			
F	5′-TTGGTTTGGTTGTGTCTCCA-3′	60	875
R	5′-ATACCAGGCCGCATAAACAG-3′		
Pair 5			
F	5′-CTCCATCATGCACTGGACAC-3′	60	920
R	5′-CCAACTGTTGTTGCTCCAGA-3′		
Pair 6			
F	5′-GCCACATCTTCTGGCAAAGT-3′	55	979
R	5′-TGAGTACCACAGCACCCAAA -3′		
Pair 7			
F	5′-GAGACCAGACTGCAAGACCAG-3′	55	698
R	5′-ACTTGCTCGTTCGTGGTAAGA-3′		
Pair 8			
F	5′-GCAGAGTGCACATACCTGGA-3′	60	704
R	5′-TGGAAGTGGAAGTGGGAGTC-3′		
Pair 9			
F	5′-AACAGGACCTTCCACGTAGC-3′	60	896
R	5′-TGTGCTGTCCAGAATGGAGT-3′		
Pair 10			
F	5′-CACAAGCCACTCCACCAGTA-3′	60	429
R	5′-CGCGGAGGTTGTAAGTTCTC-3′		

### Quantitative real-time PCR (qRT-PCR)

Total RNAs of tissues (brain, testis, heart, kidney, liver, intestine, eye, gills, tail and muscle) pooled from five 10 month old male fish, 30 embryos from 1 hpf, 5 hpf, 10 hpf, 18 hpf, 24 hpf, 48 hpf, 72 hpf and 5 days post-fertilization (dpf) timepoints, 8 larvae from 15 dpf timepoint and 8 juvenile zebrafish from 35 dpf timepoint were isolated using a Trizol reagent (15596018, Ambion, USA) and a homogenizer (Bullet Blender, Next Advence, Storm 24). DNase treatment was performed after total RNA isolation (AM1907, Turbo DNA free, Ambion, USA). For cDNA synthesis, 500 nanogram (ng) of DNase-treated total RNA was used and the manufacturer’s instructions were followed (05081955001, Transcriptor High Fidelity cDNA Synthesis Kit, Roche, Germany).

All qRT-PCR experiments were performed with the Roche Light-Cycler 480 System. cDNAs of the samples from ten tissues and ten developmental stages were diluted to a 1:4 ratio and 5 microliter (µl) were used per reaction to quantify spatial and temporal expression of zebrafish *wdr81*, respectively. Primers and probes were designed and ordered according to the Universal Probe Library Assay Design Center specific for zebrafish transcripts (Table 2). 400 nanomolar (nM) of each primer and 200 nM of each probe were used in reactions. Reactions were performed in 20 µl volumes. Each reaction was performed in duplicate on each plate and repeated 3 times. Negative control samples that did not include reverse transcriptase during cDNA synthesis and no template negative control samples were also used. Reaction conditions were 10 min at 95 °C; 10 s at 95 °C, 30 s at 60 °C, 1 s at 72 °C for 45 cycles; and 30 s at 40 °C. Ct values were obtained from LCS480 software (Roche, Germany). 2^−ΔΔCt^ method was used to calculate fold changes. ΔCt formula was applied by substracting β-actin Ct values from *wdr81* Ct values per reaction. Temporal relative expression values were calculated according to the ΔCt of 15 dpf larva and spatial relative expression values were calculated according to the ΔCt of brain. Graphics, which represent relative expression value + standard error (SE), were drawn with GraphPad [10].

**Table 2:** Primer sets to quantify temporal and spatial expression of zebrafish *wdr81*

Gene	Primers	Sequence	Probes
*wdr81*	F	5′-TCTCATGCAGGGAGTATCACA-3′	Probe 46 (cat. no. 04688066001, Roche)
	R	5′-AGGTGTCTGCTCAACGGAAT-3′	
β-Actin	F	5′-GCCTGACGGACAGGTCAT-3′	Probe 104 (cat. no. 04692225001, Roche)
	R	5′-ACCGCAAGATTCCATACCC-3′	

### Whole mount in situ hybridization (WMISH)

The spatial and temporal distribution of *wdr81* was determined using whole mount in situ hybridization. The region between primer pair 5 (forward) and primer pair 6 (reverse) from Table 1, which is the corresponding region to the mutation site in patients, was amplified from 24 hpf embryo cDNA under the following PCR conditions: 2 min at 95 °C, 30 s at 95 °C, 30 s at 62 °C, 2 min at 72 °C for 35 cycles; and 7 min at 72 °C. The amplicon was run on 0.8 % agarose gel, the observed band was extracted from the gel (D4007, Zymoclean Gel DNA Recovery Kit, USA), and cloned (A1360, pGEM-T Easy Vector System I, Promega, USA). Colonies were selected based on blue/white screening. The selected plasmid was linearized with double digestion of NdeI (ER0582, Fermentas) and SalI (ER0645, Thermo Scientific) to obtain antisense probe. Linearized plasmid, extracted from the gel (D4007, Zymoclean Gel DNA Recovery Kit, USA), was used as a template and antisense RNA probe was synthesized using T7 enzyme mix (AM1320, MaxiScript SP6/T7 In Vitro Transcription Kit, Ambion, USA) and DIG-labelling mix (11277073910, DIG RNA Labelling Mix, Roche, Germany). Embryos at 6, 10, 18, 24, 48, and 72 hpf were fixed in 4 % paraformaldehyde solution (FB001, Invitrogen IC Fixation Buffer, Invitrogen, USA), and after dehydration steps, they were kept in 100 % methanol until use. The protocol was performed as previously described [11] with some modifications. The images were taken with Zeiss Stereomicroscope Discovery V220 (Carl Zeiss, Germany). Images of 6, 10, 18, 24, 48 and 72 hpf embryos were taken at 72×, 130×, 105×, 61×, 50× and 46× magnifications, respectively. Head region images of 18, 48 and 72 hpf embryos were taken at 150×, 118× and 100× magnifications, respectively. Following whole-mount in situ hybridization, twenty micrometer thick transverse sections were taken from the head regions of 18, 48, and 72 hpf embryos using a cryostat (CM 1850, Leica) and visualized with a brightfield upright microscope (Fluorescent and DIC equipped upright microscope, Zeiss, Germany).

### In situ hybridization

The distribution of *wdr81* expression in adult brain and eye was determined using in situ hybridization. Ten micrometer thick coronal sections were taken from the eye and brain of a 10 month old male fish using a cryostat (CM 1850, Leica, Germany). The in situ hybridization experiment was carried out as described previously [1]. The antisense probe was synthesized as mentioned previously in whole mount in situ hybridization section. The same plasmid construct was used as a template for synthesis of the sense probe. NcoI (FD0573, Thermo Fisher Scientific) and ApaI (ER1411, Thermo Fisher Scientific) were utilized for linearization of the plasmid construct and the sense RNA probe was made using the SP6 enzyme mix (AM1320, MaxiScript SP6/T7 In Vitro Transcription Kit, Ambion, USA) and DIG-labelling mix (11277073910, DIG RNA Labelling Mix, Roche, Germany). The slides were visualized with the brightfield upright microscope (Fluorescent and DIC equipped upright microscope, Zeiss, Germany).

### Rapid amplification of cDNA ends (RACE)

The rapid amplification of cDNA ends (RACE) method was employed to define the 5′ untranslated region (UTR) and 3′ UTR of *wdr81* using 24 hpf embryo and adult brain total RNAs. Total RNAs of brain tissue from three 10 months old male fish and forty 24 hpf embryos were isolated with a RNeasy Mini Kit (74104, Qiagen, Germany). DNase treatment was performed with a RNase-free DNase Set (79254, Qiagen, Germany).

### Characterization of the 5′RACE Product

The GeneRacer^TM^ Kit (L1500-01, RLM-RACE, Invitrogen, USA) was employed to perform the RACE experiments. All RACE assays were carried out according to the instructions of the manufacturer. Briefly, for the 5′RACE experiment, total RNA samples of pooled 24 hpf embryos and 10 months old male fish brains were treated with calf intestinal phosphatase (CIP) and tobacco acid pyrophosphatase (TAP). Then the GeneRacer™ RNA Oligo was ligated to mRNAs in order to obtain a known priming site at the 5′ end. The GeneRacer™ RNA Oligo ligated mRNAs were converted to RACE ready first-strand cDNAs by using Cloned AMV reverse transcriptase and the GeneRacer™ Oligo dT primer. The GeneRacer™ 5′ primer (5′-CGACTGGAGCACGAGGACACTGA-3′) and the gene specific primer wdr81_Racer-5E2 (5′-ACAGTTTCTGCAGGGCTTGACGAAC-3′), which was designed according to Ensembl (ENSDART00000156621), were used to amplify the 5′RACE product of *wdr81* by touch down PCR. Touch down PCR was performed under the following conditions: 30 s at 94 °C, 30 s at 94 °C, 40 s at 72 °C, 45 s at 68 °C for 5 cycles; 30 s at 94 °C, 40 s at 71 °C, 45 s at 68 °C for 5 cycles; 30 s at 94 °C, 40 s at 70 °C, 45 s at 68 °C for 25 cycles; and 5 min at 68 °C. The touch down PCR amplicons of embryo and brain samples were used as templates for nested PCR. Primers were the GeneRacer™ 5′ nested primer (5′-GGACACTGACATGGACTGAAGGAGTA-3′) and the gene specific nested primer wdr81_Racer-5E2_Nested (5′-CTGCATATGGCTGCACATGAGTC-3′), which was designed according to Ensembl (ENSDART00000156621). Nested PCR was performed under the following conditions: 30 s at 94 °C; 30 s at 94 °C, 40 s at 72 °C, 50 s at 68 °C for 30 cycles; and 5 min at 68 °C. Resulting amplicons were run on a 1 % agarose gel, extracted from the gel (K220001, PureLink Quick Gel Extraction and PCR Purification Combo Kit, Invitrogen, USA) and cloned (45-0071, Topo TA Cloning Kit for Sequencing, Invitrogen, USA). Colonies were selected based on both ampicillin and kanamycin resistance and plasmids were isolated (K2100-11, Purelink Quick Plasmid Miniprep Kit, Invitrogen, Germany). The plasmids including the insert were sent for Sanger sequencing. Results of Sanger sequencing were analyzed with CLCBio Main Workbench software package (CLCBio Inc).

### Characterization of the 3′RACE Product

For the 3′RACE experiment, the RACE ready first-strand cDNAs were synthesized from total RNA samples of pooled 24 hpf embryos and 10 months old male zebrafish brains by using Cloned AMV reverse transcriptase and the GeneRacer™ Oligo dT primer. Using the GeneRacer™ Oligo dT primer provides a known priming site at the 3′ end of the resulting cDNA. Three overlapping amplicons as 3′RACE product of *wdr81* were aimed to be obtained. The 3 primer pairs used in this experiment are shown in Table 3. No template negative controls were included in the 3′ UTR characterization experiments.

**Table 3:** Primer pairs used in the characterization of 3′ end of *wdr81*

	Primer	Primer sequence	Expected amplicon size (bp)
Primer pair 1	F (Pair1F_Drwdr81_3RACE)	5′-CTGACAACGGTGCCATCAGG-3′	717
	R (Pair1R_Drwdr81_3RACE)	5′-TTCAGGACCATCCCATTGCATA-3′	
Primer pair 2	F (Pair2F_Drwdr81_3RACE)	5′-CTGTATCCACGTCAATGGAGCGTAA-3′	757
	R (Pair2R_Drwdr81_3RACE)	5′-GAAGCATTGTTCAATGTACGTTCGGTA-3′	
Primer pair 3	F (Pair3F_Drwdr81_3RACE)	5′-CATTTATGGTTCGCTAATTCCCTCAA-3′	634
	R (GeneRacer™ 3′ Primer)	5′-GCTGTCAACGATACGCTACGTAACG-3′	

The PCR conditions using primer pair 1 in Table 3 were 40 s at 98 °C; 10 s at 98 °C, 30 s at 68 °C, 35 s at 72 °C for 30 cycles; and 5 min at 72 °C; using primer pair 2 in Table 3 were: 40 s at 98 °C; 10 s at 98 °C, 30 s at 64 °C, 40 s at 72 °C for 30 cycles; and 5 min at 72 °C; using primer pair 3 in Table 3 were: 2 min at 95 °C; 30 s at 95 °C, 30 s at 61 °C, 60 s at 72 °C for 30 cycles; and 7 min at 72 °C. Amplicons were directly sent to Sanger sequencing after checking for the presence of the single bands on a 1 % agarose gel. Results of Sanger sequencing were analyzed with the CLCBio Main Workbench software package (CLCBio Inc).

The experiment was designed based on the predicted sequence of *wdr81*-001 (ENSDART00000156621), and the PCR product obtained with primer pair 2 in Table 3 was found to be longer than the expected size. The insertion sequence within the amplicon, which was obtained with primer pair 2 in Table 3, was further analysed by amplifying the same RACE ready cDNAs with a new primer pair. The new primer pair was designed to obtain the insertion site in a shorter frame. The sequence of the forward primer was 5′-CATTATTATCTCCAGACATTCCAA-3′ and the sequence of the reverse primer was 5′-TGAGGGAATTAGCGAACCAT-3′. The PCR conditions using this primer pair was 40 s at 98 °C; 10 s at 98 °C, 30 s at 58 °C, 30 s at 72 °C for 30 cycles; and 5 min at 72 °C. The experiment was designed to obtain a 250 bp long amplicon when the predicted sequence is present and there is no insertion site. The amplicons obtained from 24 hpf embryo and brain samples were cloned (A1360, pGEM-T Easy Vector System I, Promega, USA), colonies were selected based on blue/white screening. Plasmids were isolated (K2100-11, Purelink Quick Plasmid Miniprep Kit, Invitrogen, Germany) and 3 plasmids per sample were sequenced. Sequences were analyzed with CLCBio Main Workbench software package (CLCBio Inc).

cDNAs of 10 different adult tissues and 10 different development stages were also amplified with the same primer pair to test the presence of the insertion site. These cDNAs were amplified with a beta-actin primer pair, whose forward primer was 5′-ATTGCTGACAGGATGCAGAAG-3′ and reverse primer was 5′-GATGGTCCAGACTCATCGTACTC-3′ [12] in order to test the presence and integrity of the cDNAs. The amplicons were run on a 1 % agarose gel. The intensity of the bands were measured using Image J [13].

### Bioinformatics analysis

In order to determine the conserved domains of WDR81 protein in human, mouse and zebrafish, we aligned and compared the amino acid sequences from 3 species. The amino acid sequences encoded by the human *WDR81* (ENST00000409644), mouse *Wdr81* (ENSMUST00000173320) and zebrafish *wdr81* (ENSDART00000156621) were aligned using Clustal Omega [14] with default parameters. The alignment output file was submitted to ESPript 3 software [15]. BEACH and WD40 domain predictions of the SMART database [16] and MFS domain prediction (CLC Main Workbench software package, CLC Bio Inc) were highlighted on the ESPript 3 output file. The transmembrane domain of the zebrafish wdr81 protein was predicted by TMpred software [17].

## Results

### Zebrafish orthologue of WD repeat containing protein 81

**Fig. 1:**
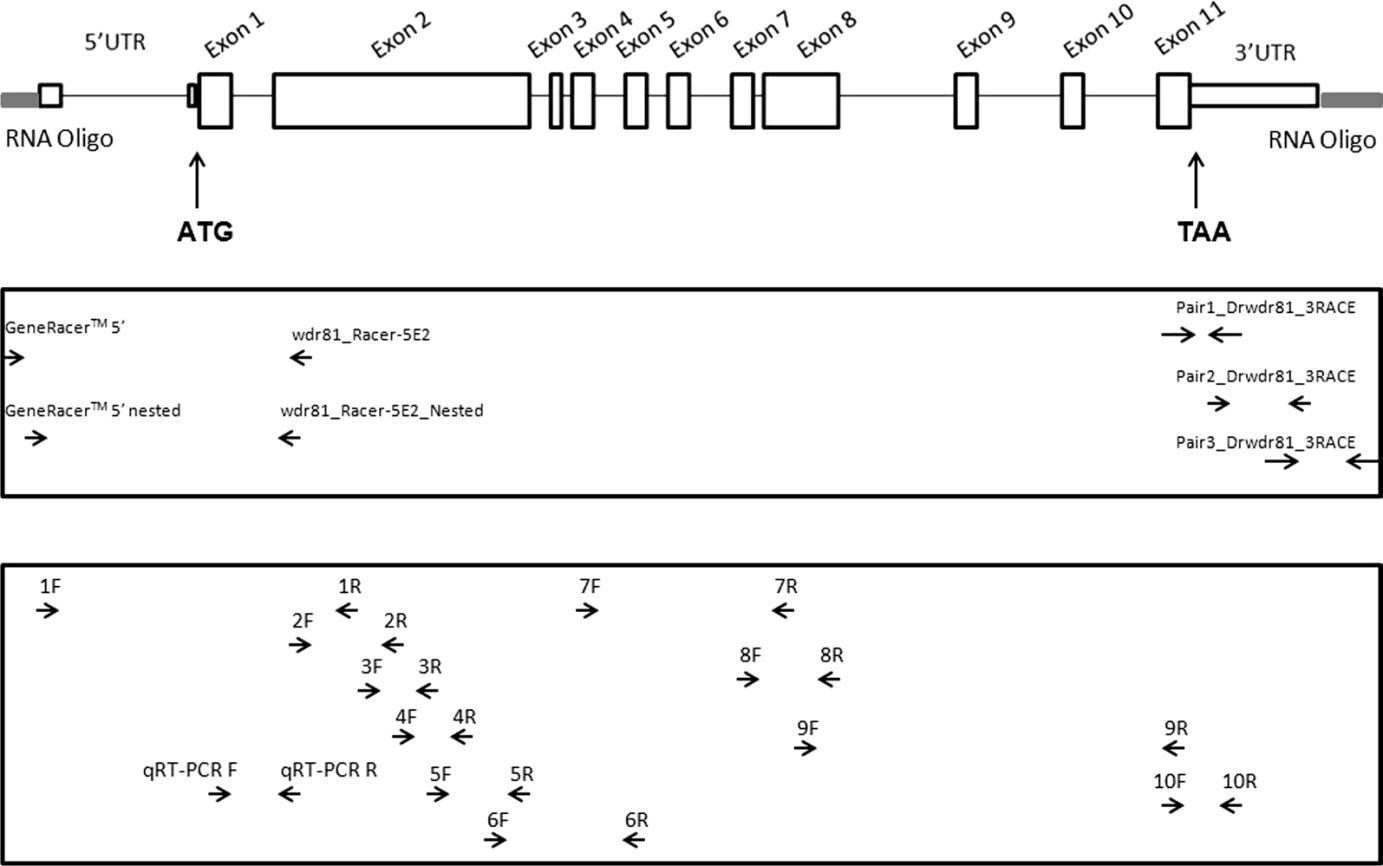
Genomic structure of the zebrafish *wdr81* and location of primers. The genomic structure of zebrafish *wdr81* was derived from the predicted sequence information released by the Ensembl database and the open reading frame was amplified based on this information. Exons are shown as *boxes* and introns are shown as *lines*. Location of RNA oligos, ligated to the transcript by employing the RACE kit are shown in *gray boxes*. *Arrows* in the *top box* illustrate the approximate binding sites of the primers used in the RACE experiments. *Arrows* in the *lower box* indicate the approximate binding sites of primers used to amplify the open reading frame and of qRT-PCR primers

Phylogenetic analysis has shown that the WD repeat containing protein 81 is highly conserved among vertebrates [1]. The zebrafish wdr81 putative protein shares 56.94 % identity with human WDR81 and 56.68 % identity with mouse Wdr81 proteins based on the Clustal Omega alignment [14]. The zebrafish *wdr81* transcript (*wdr81*-001, ENSDART00000156621) is composed of twelve exons including predicted sequences of the 5′ UTR, open reading frame and 3′ UTR (Fig. 1). This novel transcript is 8249 base pairs (bp) in length and its putative protein product is made up of 2065 amino acid residues. There is one copy of *wdr81* in the zebrafish genome and it is located on chromosome 15. It has been shown that orthologues of *wdr81* exist in other fish genomes. Spotted gar (*Lepisosteus oculatus*), Amazon molly (*Poecilia formosa*), Fugu (*Takifugu rubripes*), Medaka (*Oryzias latipes*), Tetraodon (*Tetraodon nigroviridis*), Tilapia (*Oreochromis niloticus*), platyfish (*Xiphophorus maculatus*), cave fish (*Astyanax mexicanus*), stickleback (*Gasterosteus aculeatus*) and cod (*Gadus morhua*) possess wdr81 orthologues with a percentage of identity in protein sequences ranging between 63 and 79 % [18]. Taken together these data demonstrate that *wdr81* is conserved among vertebrates, including a variety of different fish species.

Zebrafish wdr81 is predicted to be a transmembrane protein, including BEACH, MFS and WD40 repeat domains based on TMpred software, SMART database and CLC Main Workbench software predictions, which are consistent with the domain predictions of human WDR81 and mouse Wdr81 proteins (Additional file 1: Figure S1) [1, 5]. We aligned the putative protein sequence of zebrafish wdr81 with human and mouse orthologous proteins using Clustal Omega, and submitted the resulting alignment file to ESPript 3 software. We highlighted the positions of the predicted domains of human, mouse and zebrafish WD repeat containing protein 81. The most conserved domain among the three species was the BEACH domain, located between amino acid residues 337–598 of zebrafish wdr81. The MFS domain was predicted to take place between amino acid residues 951–1513 while seven WD40 repeats were predicted to reside between amino acid residues 1758–1797, 1807–1844, 1850–1889, 1892–1936, 1939–1977, 1980–2017 and 2027–2065 of zebrafish wdr81. There existed a difference in the number of WD40 repeats among the three species, one more WD40 repeat domain was predicted in zebrafish when compared to human and mouse (Additional file 2: Figure S2). Six membrane spanning domains of the zebrafish wdr81 protein were predicted to be located between amino acid residues 665–686, 1031–1055, 1413–1435, 1463–1483, 1690–1712 and 1920–1943 (Additional file 1: Figure S1).

### Zebrafish *wdr81* encodes one open reading frame

**Fig. 2:**
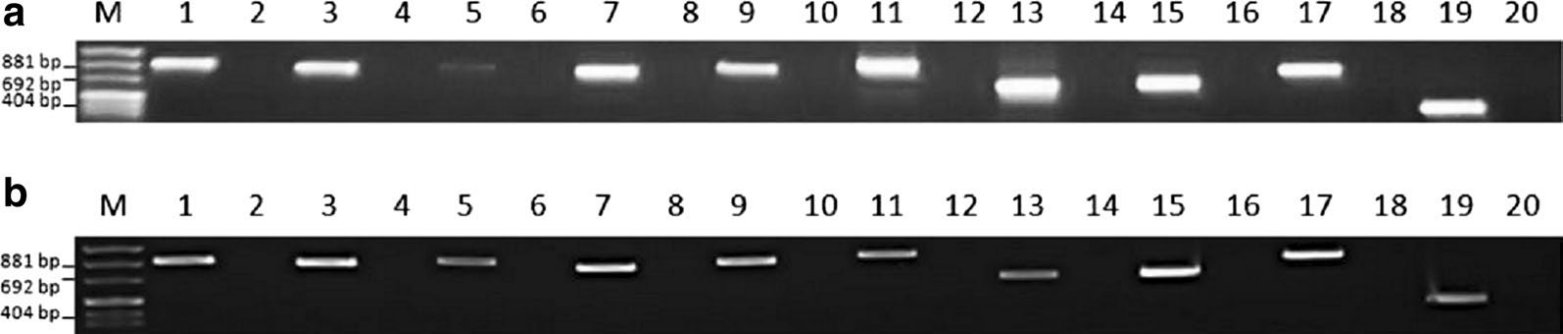
Agarose gel electrophoretic separation of overlapping amplicons of the zebrafish *wdr81* open reading frame. Amplification of the open reading frame from 24 hpf embryo cDNA and adult brain cDNA resulted in one amplicon per reaction indicating that there is one open reading frame of zebrafish *wdr81*. Since the experiment was designed based on the predicted sequence, this result also confirms the prediction. **a** Amplification of 24 hpf embryo cDNA. **b** Amplification of adult brain cDNA. *Lanes* *1, 3, 5, 7, 9, 11, 13, 15, 17* and *19* were loaded with the PCR products of primer pairs 1–10, respectively. *Lanes 2*, *4, 6, 8, 10, 12, 14, 16, 18* and *20* were loaded with the PCR products of −RT controls amplified with primer pairs 1–10, respectively. *Lanes* indicated as *M* are loaded with pUC mix DNA Marker (SM0301, Thermo Scientific)

According to the Ensembl database, human *WDR81* gene has nine known protein coding transcripts and the mouse *Wdr81* gene has three known protein coding transcripts. The zebrafish *wdr81* gene has one protein coding transcript, wdr81-001 (ENSDART00000156621). Since the human and mouse genes were predicted to have more than one transcript [1, 5, 18], we aimed to fully characterize the structure of the *wdr81* transcript and test the presence of transcript variants in zebrafish. Initially, we amplified the open reading frame (ORF) of *wdr81*. We used 24 hpf embryos as a sample from an early development stage and brain as an adult tissue sample. Our experimental design included employing ten primer pairs, which span the predicted open reading frame, in order to amplify it as ten overlapping amplicons (Fig. 1). PCRs in which 24 hpf embryo and brain cDNAs were amplified with these primer pairs resulted in one amplicon per reaction, indicating that zebrafish *wdr81* encodes one open reading frame (Fig. 2).

## 5 and 18 hpf are critical timepoints of development regarding *wdr81* expression

**Fig. 3:**
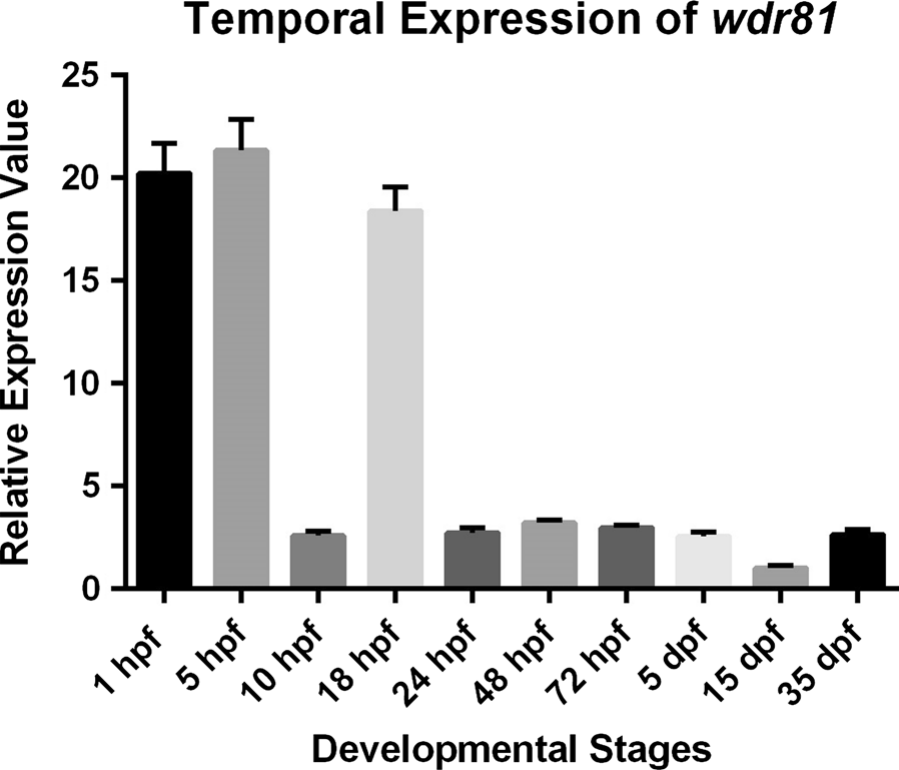
Relative temporal expression of *wdr81* as determined by qRT-PCR. (1) 1 hpf embryo, (2) 5 hpf embryo, (3) 10 hpf embryo, (4) 18 hpf embryo, (5) 24 hpf embryo, (6) 48 hpf embryo, (7) 72 hpf larva, (8) 5 dpf larva, (9) 15 dpf larva, (10) 35 dpf juvenile zebrafish. *Error bars* represent +SE

qRT-PCR revealed the temporal expression of *wdr81*. We quantified and compared the relative expression values of embryos at ten developmental stages using the primers mentioned as qRT-PCR F and R (Fig. 1). Expression of *wdr81* at 1, 5 and 18 hpf timepoints was higher than the expression at 10 hpf, 24 hpf, 48 hpf, 72 hpf, 5 dpf, 15 dpf and 35 dpf timepoints (Fig. 3).

Zygotic expression first starts at 3 hpf in zebrafish [19, 20]. Relative expression analysis of ten developmental stages demonstrated that expression of *wdr81* was high both before and after zygotic expression starts, showing that *wdr81* was maternally supplied. Expression level decreased dramatically at 10 hpf and increased sharply at 18 hpf. It decreased again at 24 hpf before being maintained during the rest of the developmental period (Fig. 3). Therefore, regarding *wdr81* expression, 5 and 18 hpf timepoints were found to be critical developmental stages after zygotic expression started.

**Fig. 4:**
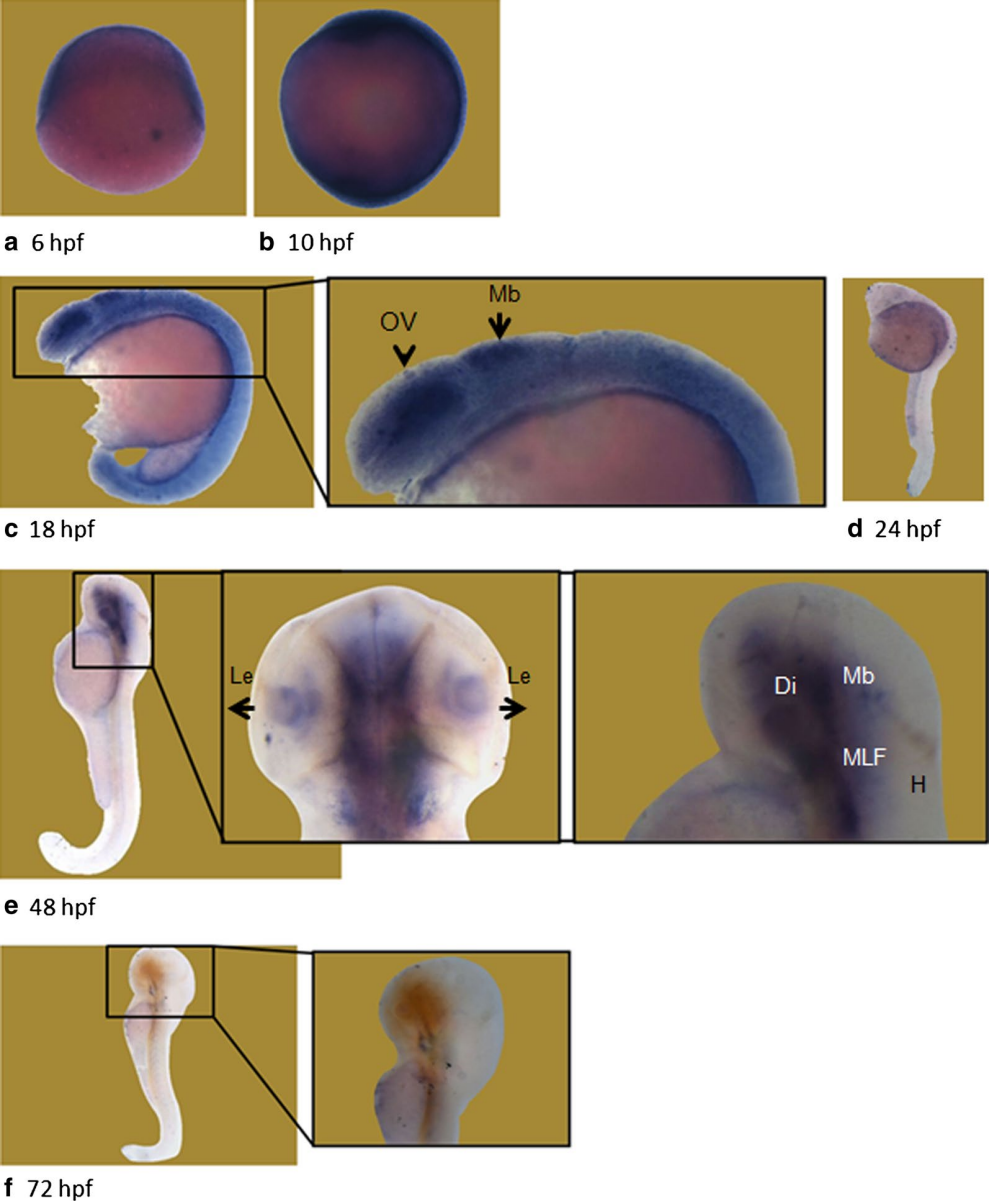
Whole mount in situ hybridization revealed differential expression of *wdr81* transcript during embryonic development. Our results from the WMISH method are in parallel with the qRT-PCR data. The signal is high during the first 3 developmental timepoints (6–18 hpf), it is decreased and maintained during the rest of the development periods (24–72 hpf). *OV* optic vesicle, *Mb* midbrain, *Le* lens, *H* hindbrain, *Di* diencephalon, *MLF* medial longitudinal fascicle

Our whole mount in situ hybridization results were also consistent with the results of the qRT-PCR. A riboprobe detecting a 1.7 kb region between primers 5F-6R (Fig. 1) of the *wdr81* ORF was used and this region includes the sequence which corresponds to the mutation site in human patients. The intensity of the signal weakened after the 18 hpf timepoint. We observed that signals obtained from *wdr81* RNA probe were high at the early developmental stages (6–18 hpf), then decreased before being maintained at low levels during the rest of the evaluated timepoints (24–72 hpf). Signals were relatively high in the head at 18 and 48 hpf timepoints (Fig. 4).

### *wdr81* is ubiquitously expressed and enriched in the eye and brain

Our whole mount in situ hybridization study in which *wdr81* expression at six embryonic stages was investigated, also showed ubiquitous expression at 6, 10, 18, and 24 hpf timepoints. The signal was stronger in the optic vesicle (OV) and the midbrain (Mb) at the 18 hpf timepoint. Moreover, the signal was condensed in the lens (Le), diencephalon (Di), midbrain (Mb), and medial longitudinal fascicle (MLF) at 48 hpf. Finally, the signal dramatically decreased at 72 hpf timepoint (Fig. 4).

**Fig. 5:**
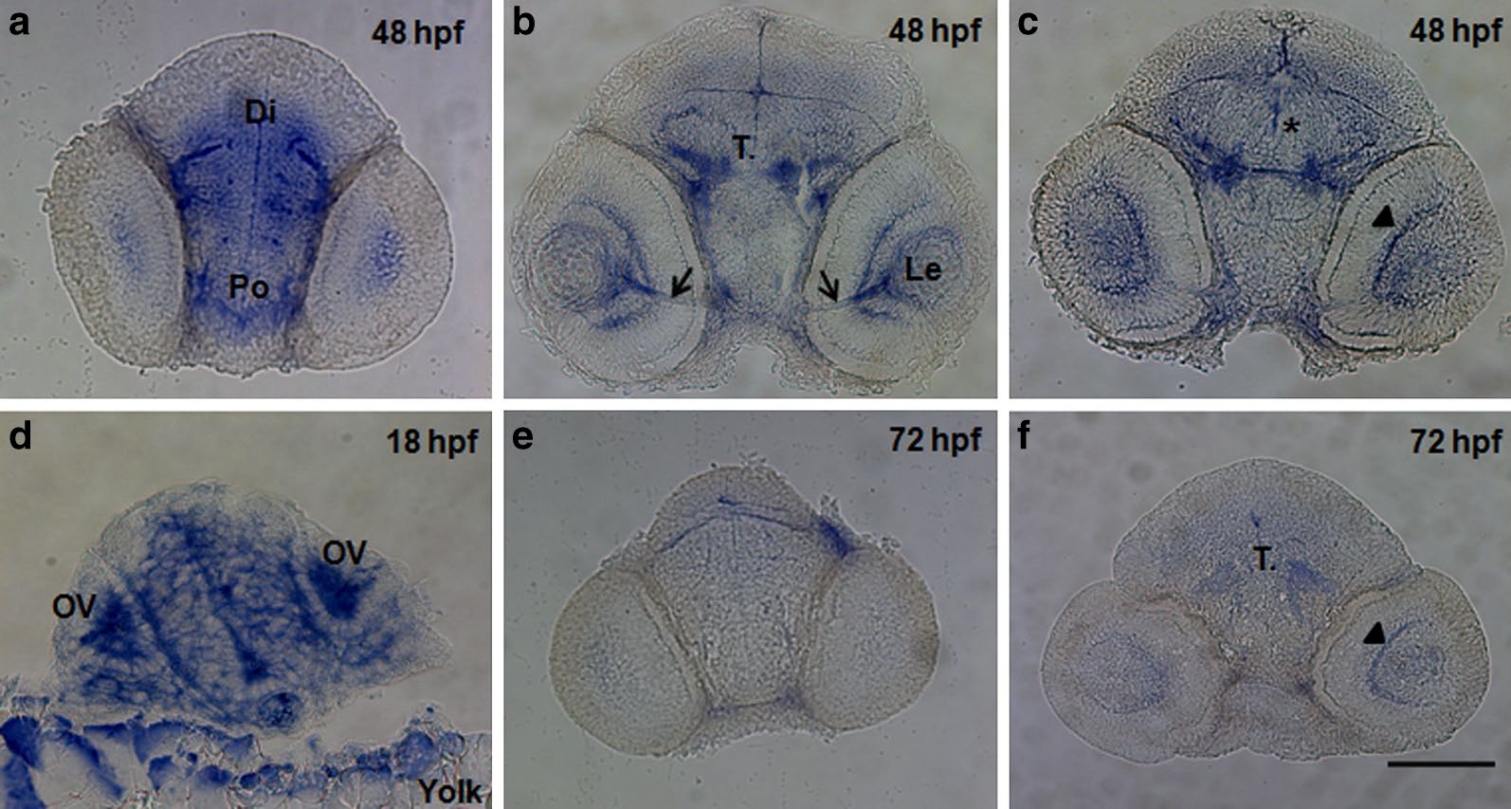
Transverse sections through the head regions of whole mount in situ hybridization specimens at 3 embryonic timepoints. The expression of *wdr81* was observed in a regionally-specific manner by 48 hpf (**a**–**c**), whereas it was ubiquitously expressed at 18 hpf (**d**), and decreased by 72 hpf (**e**, **f**). *Arrows* indicate the optic nerve, *asterisk* the region of the nucleus of the medial longitudinal fascicle, and *arrowhead* the retina. *Po* preoptic area, *Di* diencephalon, *T.* midbrain tegmentum, *Le* lens, *OV* optic vesicle, *Yolk* yolk sac. *Scale bar* equals 100 μm

In order to further identify more specifically the brain and eye regions expressing *wdr81*, we sectioned the head regions of our whole mount in situ embryos from the 18, 48 and 72 hpf timepoints into 20 μm-thick sections. Our data indicated that the highest level of expression was observed at 18 hpf and uniformly distributed throughout the optic vesicle and brain (Fig. 5d) likely indicating that *wdr81* is found in all cell types. At 48 hpf, a more select pattern of staining is observed. *wdr81* gene expression is found in areas which included the preoptic area (Po), diencephalon (Di), midbrain tegmentum (T), optic nerve, lens, nucleus of the MLF, and the retina. This reduced and more specific pattern, including an association with axonal fibers, likely indicates a neuronal phenotype (Fig. 5a–c). The *wdr81* expression was very reduced by 72 hpf with staining observed in the retina and midbrain tegmentum (T) (Fig. 5e–f).

**Fig. 6:**
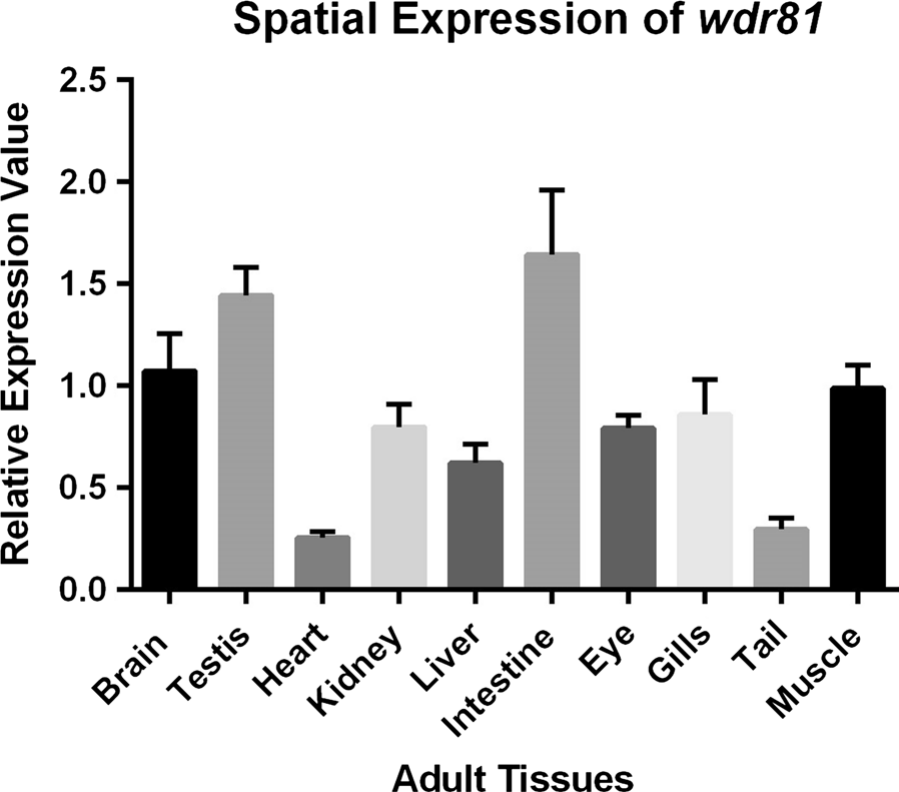
Relative spatial expression graphic of *wdr81* as determined by qRT-PCR. (1) Brain, (2) testis, (3) heart, (4) kidney, (5) liver, (6) intestine, (7) eye, (8) gills, (9) tail, (10) muscle. *Error bars* indicate +SE

We investigated the spatial expression of *wdr81* in adults using the same primer pair in the temporal expression study by employing the qRT-PCR method. For this analysis 10 different adult tissues were used and these tissues included brain, testis, heart, kidney, liver, intestine, eye, gills, tail, and muscle. Our results indicated that the relative expression of *wdr81* was ubiquitous, however, there were differences in the levels of *wdr81* expression among tissues (Fig. 6).

**Fig. 7:**
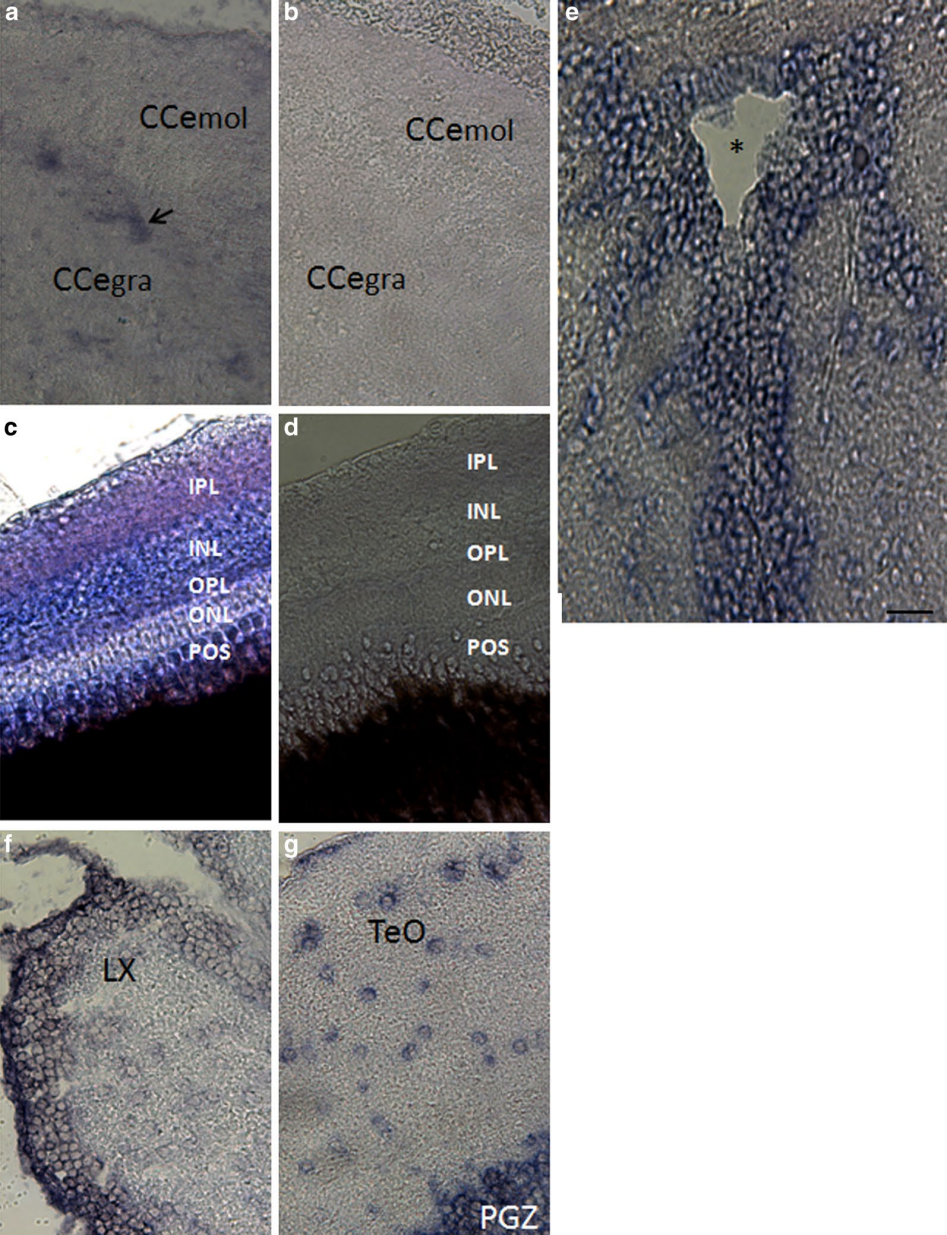
*wdr81* expression in the adult brain and eye tissues. *wdr81* expression was detected in the cerebellum (**a**), retina (**c**), tectal ventricle (**e**), brain stem (**f**), and optic tectum (**g**). Results with a sense probe in both cerebellum (**b**) and retina (**d**), which demonstrate no staining, indicates the specificity of the signal obtained with an antisense probe and both cerebellum (**b**) and retina (**d**) are shown. *CCe*_*mol*_ Cerebellar molecular layer, *Cce*_*gra*_ cerebellar granular layer, *POS* photoreceptor outer segments, *ONL* outer nuclear layer, *OPL* outer plexiform layer, *INL* inner nuclear layer, *IPL* inner plexiform layer, *LX* *lobus vagus*, *TeO* optic tectum, *PGZ* periventricular *gray* zone of the optic tectum. The *arrow* indicates the Purkinje cell layer and the *asterisk* the tectal ventricle. *Scale bar* equals 200 μm

In situ hybridization studies were performed in adult brain and eye tissues in order to further understand the expression pattern of *wdr81* within these regions. Our data demonstrated that although *wdr81* is found throughout the zebrafish brain and eye tissues, there is regional specificity. The *wdr81*-positive cells in the brain appeared to be morphologically similar to neurons and there were *wdr81*-positive fibers (Fig. 7). In the cerebellum we observed expression in presumptive Purkinje cells and in layers of the retina (Fig. 7a, c). Positive cells that are presumptive neurons were also found in multiple areas of the brain including the *lobus vagus* and optic tectum (Fig. 7f, g). Interestingly we also observed a strong expression pattern in proliferation zones of the zebrafish brain including midline ventricle, which is known as the tectal ventricle, *lobus vagus* and periventricular gray zone of optic tectum (Fig. 7e–g). Thus, our data indicate that in the adult brain there is an association of *wdr81* with areas involved in neurogenesis and based on the morphology they are likely neuronal in nature.

### Characterization of 5′ UTR of *wdr81* confirmed the predicted sequence and characterization of 3′ UTR indicated a possible alternative polyadenylation process among tissues

**Fig. 8:**
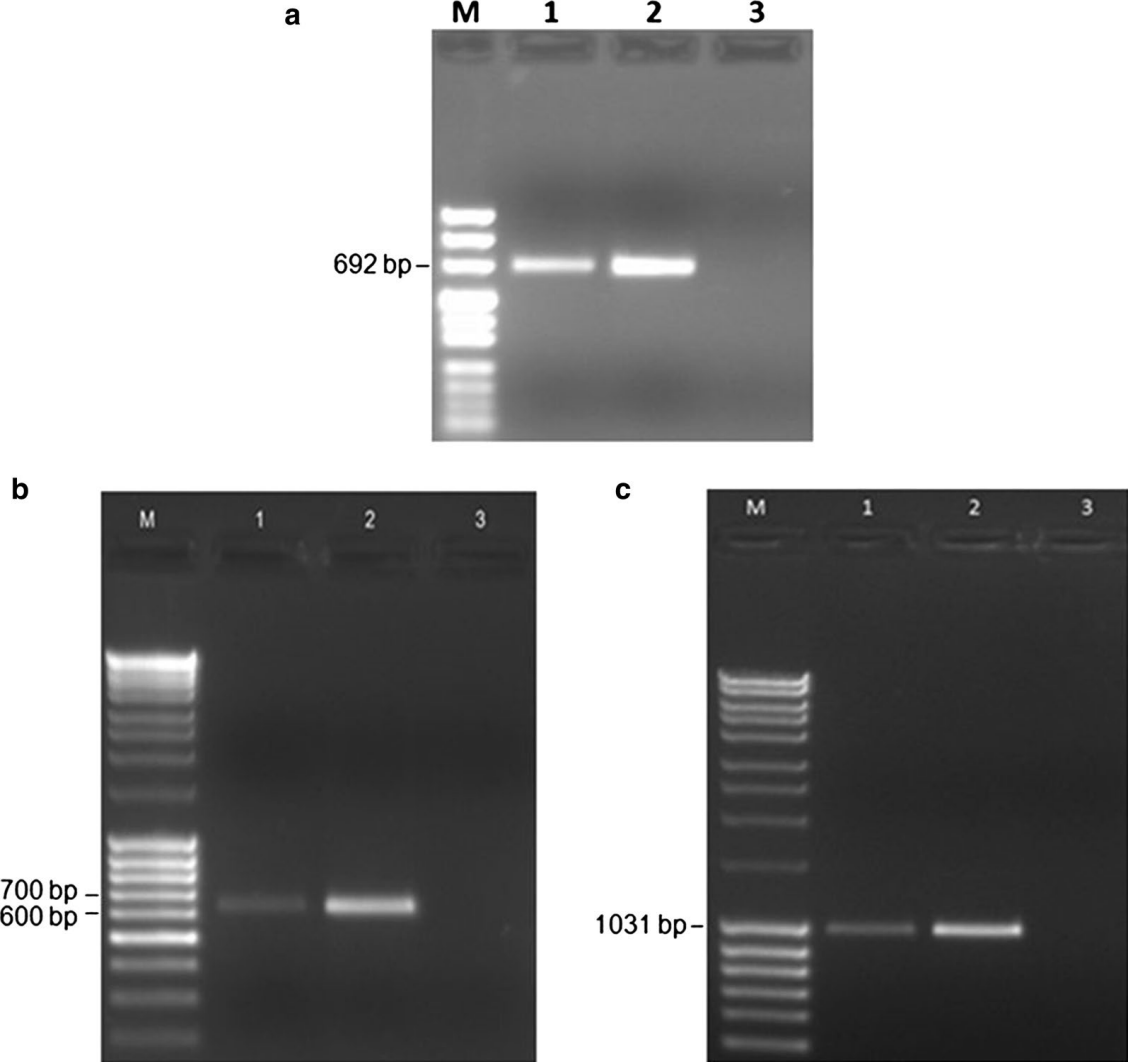
Agarose gel electrophoretic separation of the 5′RACE and overlapping 3′RACE products of zebrafish *wdr81* transcript. **a** Expected size of the 5′RACE amplicon based on the experimental design was obtained and one amplicon was observed as a PCR product indicating that there is one 5′ UTR structure of zebrafish *wdr81*. The band of ~692 bp was excised, cloned and sequenced. *Lane 1* 5′RACE product of 24 hpf embryo *wdr81* cDNA; *lane 2* 5′RACE product of brain *wdr81* cDNA; *lane 3* negative control without template, *M* pUC mix marker 8 (SM0301, Thermo Scientific). Expected size of the overlapping 3′RACE amplicons based on the experiment design were obtained from the PCR experiments with primer pairs 1 (data not shown) and 3 from the Table 3. The resulting amplicon obtained with primer pair 2 was longer than the expected amplicon size indicating presence of an insertion site. One amplicon per reaction was obtained as PCR products concluding that there is one transcript of zebrafish *wdr81.* **b** Amplification with primer pair 3 from Table 3. **c** Amplification with primer pair 2 from Table 3. In both gels, **b** and **c**, *lane 1* 24 hpf embryo RACE ready cDNA, *lane 2* brain RACE ready cDNA, *lane 3* no template negative control. *M* MassRuler Mix DNA Marker (SM0403, Thermo Scientific)

We characterized the cDNA ends of *wdr81* in order to observe whether there were transcript variants related with the 5′ UTR and 3′ UTR sequences. We performed RACE to characterize the UTR sequences of *wdr81* and used gene specific and GeneRacer™ primers (Fig. 1). The 5′RACE product was obtained by performing nested PCR followed by touch down PCR and one amplicon per reaction was obtained with the 24 hpf embryo and brain templates (Fig. 8a). Our experimental design was aimed at obtaining the 5′RACE product as an amplicon, which included the 5′ UTR, exon 1 and initial sequences of exon 2. The resulting amplicons were cloned and sequenced. Sanger sequencing results showed that the 5′ UTR of *wdr81* was found to be 264 bp in length, which was 6 bp shorter than the predicted sequence at the 5′ end (data not shown).

The 3′RACE products were obtained as three overlapping amplicons from 24 hpf embryo and brain samples. Using our experimental design, we aimed at obtaining the 3′RACE product as amplicons including the 3′ UTR, and exon 11. One amplicon per reaction was obtained as a result (Fig. 8b, c). We observed a longer amplicon than the predicted sequence in the reaction with primer pair 2 (Table 3) and analysis of the Sanger sequencing results of the 3′RACE product indicated the presence of an insertion site in amplicon 2 (Fig. 8c). The results from Sanger sequencing of 3′RACE products, which were obtained from amplicons 1, 2 and 3, confirmed the predicted sequence along with some detected variants, except the insertion site in amplicon 2 (Additional file 3: Table S1).

**Fig. 9:**
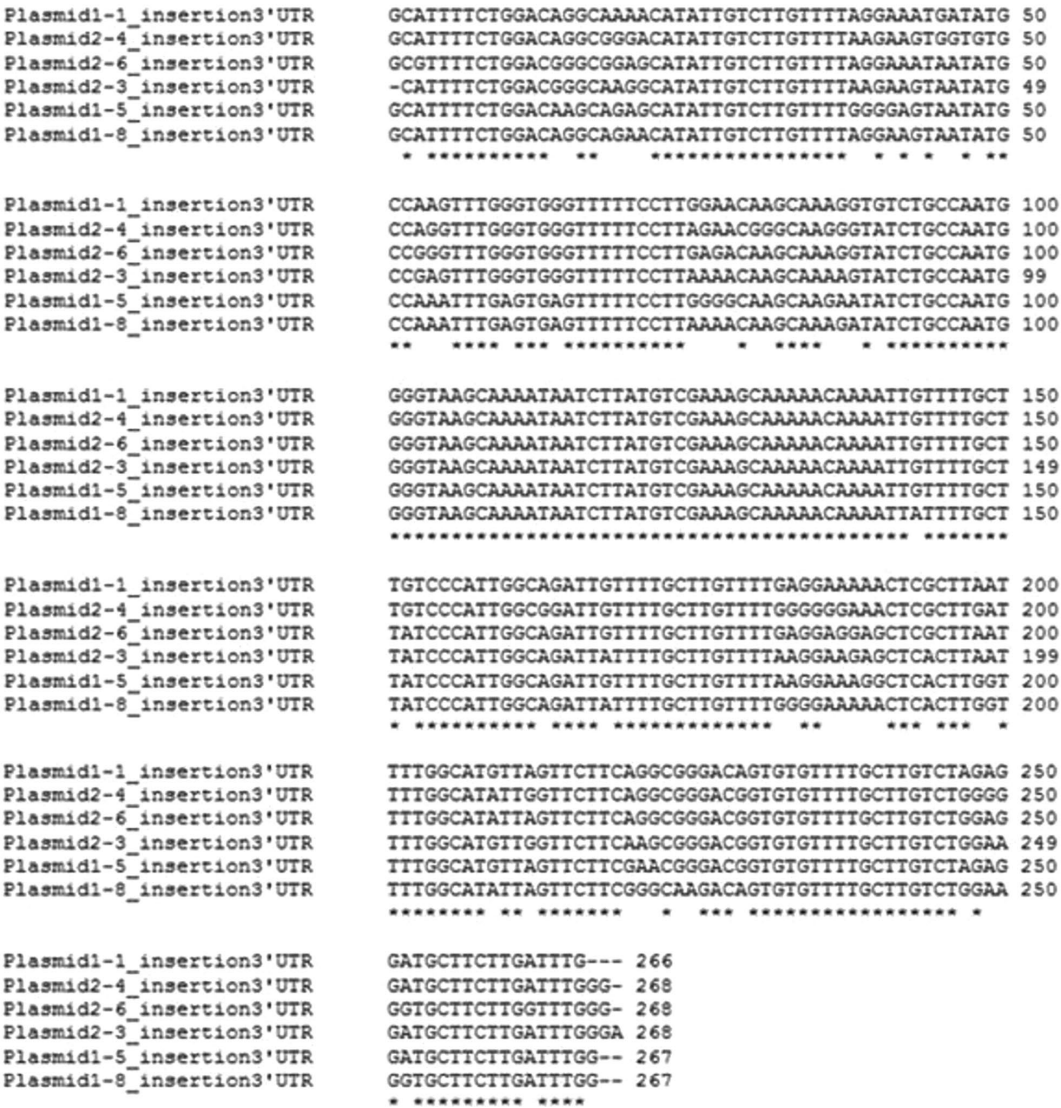
A 266 bp long insertion was determined in the 3′ UTR of the zebrafish *wdr81* transcript. Results of 3′ UTR characterization experiments indicated presence of an insertion site in 24 hpf embryo and adult brain templates. When the insertion site was further analyzed, cloned and sequenced, the sequence of the insertion site from 6 single colonies was revealed as being 266 bp in length. Twenty-four hpf embryo sample is the insert of plasmids 1–1, 1–5, and 1–8 and brain sample is the insert of plasmids 2–3, 2–4, and 2–6

**Fig. 10:**
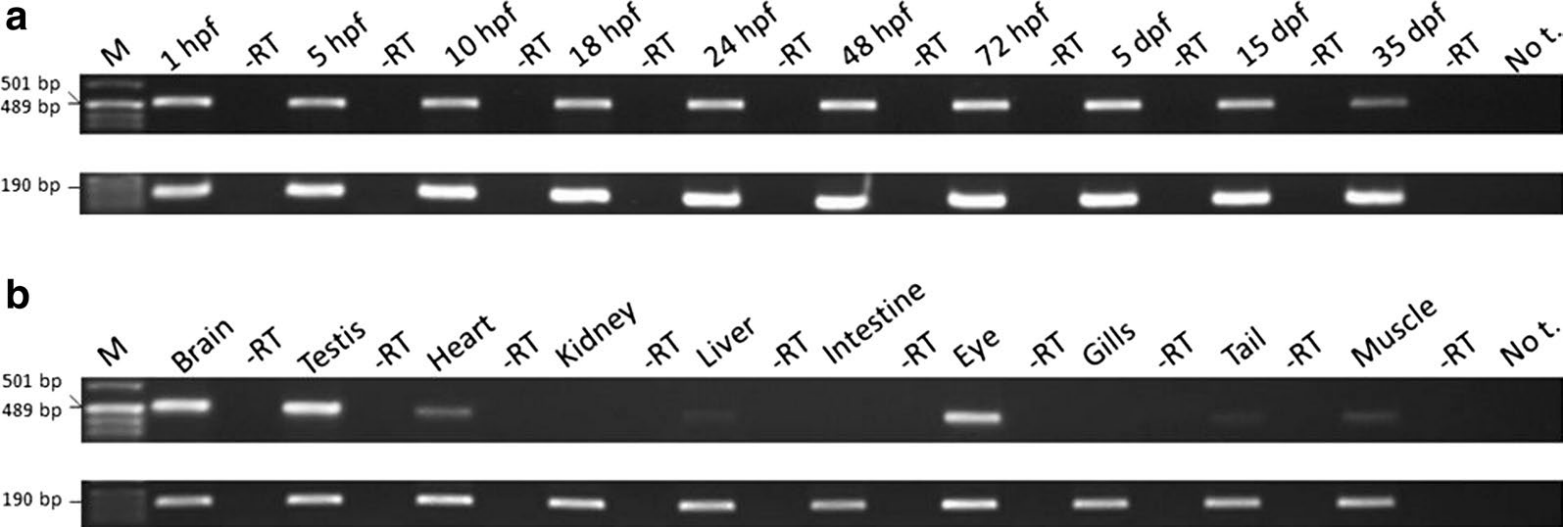
Detection of the insertion in the tested developmental and tissue samples except kidney, intestine, gills. **a** Agarose gel electrophoretic separation of the amplicons as products of a PCR experiment in which the presence of the detected insertion site in *wdr81* 3′ UTR was tested among templates from several developmental stages (*top* gel picture). Presence and integrity of the cDNA samples were tested with beta-actin amplification (*lower* gel picture). **b** Agarose gel electrophoretic separation of the amplicons as products of a PCR experiment in which the presence of the detected insertion site in *wdr81* 3′ UTR was tested among templates from several adult tissues (*top* gel picture). Presence and integrity of the cDNA samples were tested with beta-actin amplification (*lower* gel picture). In all gels, lanes were labelled with the cDNA source of each reaction loaded. *Lane* indicated as M was loaded with pUC mix DNA Marker (SM0301, Thermo Scientific). “No t.” indicates the no template negative control sample in which water was used instead of a template

The insertion site, which was observed in amplicon 2 between the nucleotides 7564 and 7565 of *wdr81* cDNA, was amplified in a shorter frame, cloned and sequenced. The alignment of the sequences from single colonies, obtained from 24 hpf embryo (plasmids 1–1, 1–5, and 1–8) and brain (plasmids 2–3, 2–4, and 2–6) templates, revealed that the insertion site was 266 bp in length (Fig. 9). Presence of the insertion site was tested in samples from several developmental stages and adult tissues. The insertion site was detected in all the tested samples of developmental stages and most of the tissues (brain, testis, heart, liver, eye, tail and muscle) however no amplicon was detected in kidney, intestine and gills (Fig. 10). The intensity of the bands in the Fig. 10 was measured (Table 4). Comparison of the intensities of the PCR products amplified from the insertion site in ten samples from different developmental stages showed that the 35 dpf juvenile zebrafish had the lowest intensity level from the insertion region. When we compared the intensities of the amplicons obtained from the insertion site in 10 adult tissues we concluded that the intensity levels of the insertion region are the highest and almost equal in the brain and testis, with the next highest intensity being observed in the eye. The intensity level of the insertion region is almost equal in heart and muscle and this level is followed by tail and liver, respectively.

**Table 4:** Band intensities of the amplicons from Fig. 10

Developmental stage	Intensities of the PCR products amplified from the insertion site	Intensities of the β-actin PCR products
(a) Band intensities of the amplicons from Fig. 10a		
1 hpf	8938.29	9821.86
5 hpf	7941.21	11,415.03
10 hpf	8265.58	13,168.54
18 hpf	8280.21	13,521.12
24 hpf	8379.09	13,497.54
48 hpf	9394.56	12,853.64
72 hpf	9327.96	13,705.19
5 dpf	9390.74	13,497.32
15 dpf	7502.30	13,005.18
35 dpf	4343.33	12,577.55

Adult tissue	Intensities of the PCR products amplified from the insertion site	Intensities of the β-actin PCR products
(b) Band intensities of the amplicons from Fig. 10b		
Brain	9475.08	7483.83
Testis	10,441.17	8470.90
Heart	2206.91	9003.5
Kidney	N/A	8421.62
Liver	407.82	8001.1
Intestine	N/A	6438.65
Eye	7023.97	9123.22
Gills	N/A	7566.79
Tail	415.84	7496.79
Muscle	1044.75	7563.46

In conclusion, analysis of the 5′RACE product revealed that *wdr81* does not have transcript variants in the 5′ UTR. Interestingly detection of the insertion site in 3′ UTR of *wdr81* indicates that there might be an alternative polyadenylation event among tissues, which results in different lengths of 3′ UTR sequences.

## Discussion

In the present study, we characterized the novel zebrafish gene, *wdr81*, a WD repeat-containing protein 81. Our study is the first report of the characterization of the *WDR81* sequence and expression patterns in zebrafish. We observed that zebrafish *wdr81* encodes one open reading frame and the transcript possesses one 5′ UTR. The sequence of 3′ UTR confirms the predicted sequence along with a 266 bp long insertion site in 24 hpf embryos and adult brain under our experimental conditions. The insertion site was also detected in testis, heart, liver, eye, tail and muscle, however it was not detected in kidney, intestine and gills. This indicates the possible presence of alternative polyadenylation processes among tissues. Five and eighteen hpf are likely to be the critical timepoints of development regarding the expression of the gene of interest because they correspond to peaks in *wdr81* gene expression levels. *wdr81* was ubiquitously expressed among tissues and enriched in the eye and brain regions in embryos, the signal of RNA probe was stronger in the eye and the midbrain at 18 hpf and was condensed in the lens, diencephalon, midbrain, and medial longitudinal fascicle at 48 hpf, which dropped down at 72 hpf. In the adult brain and eye tissues *wdr81* was expressed in presumptive neurons based on morphology and associated with neuronal proliferative zones.

Human studies have established that the missense mutation in the *WDR81* isoform 1 results in significant decreases in the volume in cerebellum and corpus callosum [1] and the neuro-ophthalmic examination of four patients with CAMRQ displayed downbeat nystagmus [21]. *WDR81* has been found to be mutated in >10 % of the colorectal cancer cell lines [4] and SERPINF2-WDR81 loci was reported to be one of the six significant loci for serum albumin as a result of transethnic meta-analysis [22]. Expression of the human *WDR81* was found to be ubiquitous and enriched in the cerebellum and corpus callosum [1]. A mouse study of a mutant line *nur5*, carrying a mutation in the predicted major facilitator superfamily domain of the Wdr81 protein, showed a similar phenotype with CAMRQ. This study revealed that Wdr81 was localized in the mitochondria of Purkinje cells and also expressed in photoreceptor cells of the adult animal [5]. The findings about WDR81 emphasize its importance in neurodevelopment, however, the exact function of the protein is not yet known. In order to provide a basis for functional studies, we aimed at characterizing *wdr81* in wild type zebrafish.

We showed that the putative zebrafish wdr81 includes the identical domains as the human and mouse orthologue proteins. The most conserved domain is BEACH domain among three species, however, there is a difference in the number of WD40 repeats among the three species, one more WD40 repeat domain is predicted to exist in zebrafish as compared to human and mouse proteins (Additional file 2: Figure S2). Conservation of wdr81 among vertebrates regarding the phylogenetic analysis [1] and domain predictions [1, 5] suggest a similar function of WDR81 in human, mouse and zebrafish.

In order to provide an experimental proof of the prediction made by Ensembl (ENSDART00000156621) and to test whether there are transcript variants in zebrafish as in mouse and human, we amplified the ORF and performed RACE experiments. We employed the RACE method to characterize the UTR sequences of *wdr81* because the UTR sequences are critical regions in post-transcriptional regulation of gene expression [23]. We concluded that zebrafish *wdr81* encodes one open reading frame (Fig. 2). The 5′ UTR of *wdr81* was found to be 264 bp in length, which is 6 bp shorter than the predicted sequence at the 5′ end. Analysis of the 5′RACE product revealed that *wdr81* does not have transcript variants regarding the 5′ UTR. The 3′RACE products were obtained as three overlapping amplicons from 24 hpf embryo and brain samples. One amplicon per reaction was obtained as a result (Fig. 8b, c). The 266 bp long insertion site detected in all the tested samples of developmental stages and most of the adult tissues (brain, testis, heart, liver, eye, tail and muscle) could not be detected in kidney, intestine and gills (Fig. 10). The intensity of the bands amplified from brain, testis and eye are the highest. Indeed, gene expression of brain and testis are highly similar in human and mouse [24]. Eyes are part of the central nervous system and a similar pattern in brain and eyes might be taking place in *wdr81* 3′ UTR. Detection of the insertion site in the 3′ UTR of *wdr81* indicates that there might be differences in the polyadenylation events among tissues, which results in different lengths of 3′ UTR sequences. Alternative polyadenylation events have been previously reported in ubiquitously expressed genes, which provide a mechanism to regulate tissue specific protein levels by changing the ratios of 3′ UTR isoforms and changing targets of microRNAs and regulatory proteins [25]. Since the insertion site in 3′ UTR might also be a target for microRNAs, we searched for potential microRNAs which target the sequence of the insertion. We found out that microRNAs 19 b and 722 might target the insertion site detected in both embryo and brain specimens [26]. The regulation of the function of the 3′ UTRs are dependent on a combination of the primary and secondary structures of the RNA sequences that form regulatory motifs [23]. Presence of the insertion site in the 3′ UTR of *wdr81* would also affect secondary structure formation [27]. Taken together, these possible mechanisms might be contributing to the regulation of different protein levels among tissues and altering the function of *wdr81*.

Our spatial expression study demonstrated that the expression of *wdr81* is ubiquitous with enriched expression in brain and eye, which is consistent with the human and mouse studies [1, 5, 21]. *wdr81* expression at six embryonic stages showed ubiquitous expression at 6, 10, 18, and 24 hpf timepoints. The signal was stronger in the optic vesicle and the midbrain at the 18 hpf timepoint. The signal was condensed in the lens, diencephalon, midbrain, and medial longitudinal fascicle at 48 hpf (Fig. 4). The medial longitudinal fascicle is the axon tract which has an important role in coordination of conjugate eye movements. The diencephalon is composed of the rudiments of the thalamus, hypothalamus and the optic cups. The optic cups give rise to the neural part of the retina. The dentate axons, which leave the cerebellum, intersect at the fiber tracts of the superior cerebellar peduncle in the posterior midbrain [28]. Taking into consideration that cerebellum and the eye regions are the common expression sites in human and mouse, our findings in developing wild type zebrafish show relevant results in the eye and brain regions.

The spatial expression studies of *wdr81* gave additional information about putative cellular phenotypes and function in addition to the regional specificity throughout the brain and eye. Our data indicated based on the expression pattern of *wdr81* in the cells of the Purkinje cell layer, retinal layers, and optic tectum, as well as being observed in the optic nerve and other fibers in the brain, that this gene is associated with multiple neuronal phenotypes in the embryo and adult. It is likely not associated with glia during development because the expression pattern is highest and ubiquitously distributed before gliogenesis begins at 22 hpf [29] and the expression pattern is decreased and more specifically localized with neuronal phenotypes at that timepoint. In the adult, the morphology of the cells appears to be neuronal in nature not glial suggesting a neuronal phenotype as well. However, future studies should be directed at determining the exact neuronal types (i.e., excitatory, inhibitory, etc.) in embryos and adults. In terms of the developmental pattern of neurogenesis in the zebrafish, it starts around 10 hpf with synaptogenesis beginning at 18 hpf [30]. Our data demonstrating that *wdr81* peaks at 18 hpf suggests that this gene maybe playing a role in continued neuronal proliferation, migration and survival in embryos. Interestingly, we observed a pattern of expression of *wdr81* in the adult that was strongly associated with proliferative zones in the zebrafish [31]. This association strongly suggests that this gene may have a role in neuronal proliferation, migration, and survival in the adult. This would be consistent with the predicted role of this gene in the brain [29, 32]. Taken together this evidence strongly suggests the function of *wdr81*.

Our temporal expression study demonstrated that the expression of *wdr81* at 1 hpf, 5 hpf and 18 hpf timepoints is higher than the expression at 10 hpf, 24 hpf, 48 hpf, 72 hpf, 5 dpf, 15 dpf and 35 dpf timepoints (Fig. 3). Zygotic expression starts at 3 hpf in zebrafish [19, 20]. Relative expression analysis of ten developmental stages demonstrated that expression of *wdr81* is high both before and after zygotic expression, indicating that *wdr81* is maternally supplied. The 5 and 18 hpf timepoints are critical developmental stages with regard to *wdr81* expression since these timepoints are after zygotic expression starts. Presumptive brain, which appears at the sphere stage (4.0–4.33 hpf) already exists at the 5 hpf timepoint and the brain structure, which appears at the segmentation stage (14–16 hpf) has been formed at the 18 hpf timepoint [29]. Our whole mount in situ hybridization data also confirmed our results of the qRT-PCR study. The intensity of the signal weakened after 18 hpf timepoint. We observed that signals obtained from *wdr81* RNA probe are high at the early developmental stages (6–18 hpf), then it decreases and is maintained at decreased levels during the rest of the evaluated timepoints (24–72 hpf). Signals are enriched in the head at 18 and 48 hpf timepoints (Fig. 4). Having the zygotic expression of *wdr81* the highest at 5 and 18 hpf and observing the signal of RNA probe high in the brain regions and eye at 18 and 48 hpf timepoints also suggests that *wdr81* might be a critical gene in neurodevelopment as reported previously [1]. Gene knock-down studies with antisense morpholinos, based on our findings reported in this study are currently being performed in order to understand the potential functional role of *wdr81* during development.

## Conclusions

In the present study, we aimed at fully characterizing the structure of the zebrafish *wdr81* transcript, detecting the presence/absence of possible transcript variants and revealing its temporal and spatial expression in zebrafish. We observed that zebrafish *wdr81* encodes one ORF, the transcript has one 5′ UTR and the sequence of 3′ UTR confirms the prediction along with a detected 266 bp long insertion site in 24 hpf embryo and adult brain. The insertion site is detected in most of the evaluated tissues, however, it was not detected in kidney, intestine and gills, which might be indicating a possible alternative polyadenylation process among tissues. *wdr81* is ubiquitously expressed among adult tissues and enriched in the eye and brain in early developmental stages. The 5 and 18 hpf are critical timepoints of development regarding *wdr81* expression corresponding to peak changes in neurogenesis. Presumptive brain and the brain structure have already formed at these timepoints, respectively. The signal of the RNA probe was stronger in the eye and the midbrain at 18 hpf and was condensed in the lens, diencephalon, midbrain, and medial longitudinal fascicle at 48 hpf, which dropped down at 72 hpf. In the adult the *wdr81*-positive cells appeared to have a neuronal phenotype based on morphology and the expression was regionally specific with an association in neurogenic regions. Taken together these data indicate the importance of the gene during neurodevelopment and adulthood with a possible role in neuronal proliferation, migration, and survival. Future studies are ongoing to determine the functional role of *wdr81*.

## Additional files

10.1186/s12868-015-0229-4 The predicted structural organization of zebrafish wdr81 protein. The putative zebrafish wdr81 protein shares a similar structure with human and mouse WD repeat-containing protein 81, which is composed of six membrane-spanning domains, BEACH, MFS and WD40 repeat domains. Human and mouse WDR81 proteins are predicted to possess six WD40 repeat domains whereas zebrafish wdr81 protein is predicted to include seven WD40 repeat domains.
10.1186/s12868-015-0229-4 Alignment of the putative wdr81 protein in zebrafish with human WDR81 and mouse Wdr81 proteins. Alignment was analyzed with ESPript program [15]. Identical amino acid residues through the proteins of the three organisms are highlighted. The boxed areas in blue indicate BEACH domain, in green indicate MFS domain and in purple indicate WD40 repeat domains. WD40 repeat domains are marked with numbers. Number 6 is predicted to exist only in zebrafish.
10.1186/s12868-015-0229-4 Variants in 24 hpf embryo and brain wdr81 3’UTR except the 266 bp long insertion.
