# Supplementary figures and images for: Characterization of a novel zebrafish (Danio rerio) gene, wdr81, associated with cerebellar ataxia, mental retardation and dysequilibrium syndrome (CAMRQ)
Source: BMC Neurosci. 2015 Dec 23;16:96. doi: 10.1186/s12868-015-0229-4 (PMC4690267; doi:10.1186/s12868-015-0229-4)

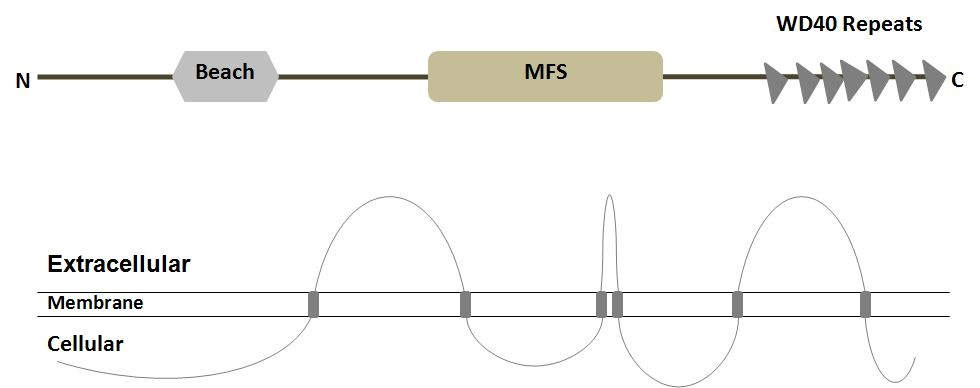

Supplement: Supplementary file 1 — 10.1186/s12868-015-0229-4 The predicted structural organization of zebrafish wdr81 protein. The putative zebrafish wdr81 protein shares a similar structure with human and mouse WD repeat-containing protein 81, which is composed of six membrane-spanning domains, BEACH, MFS and WD40 repeat domains. Human and mouse WDR81 proteins are predicted to possess six WD40 repeat domains whereas zebrafish wdr81 protein is predicted to include seven WD40 repeat domains. [file 12868_2015_229_MOESM1_ESM.tif]

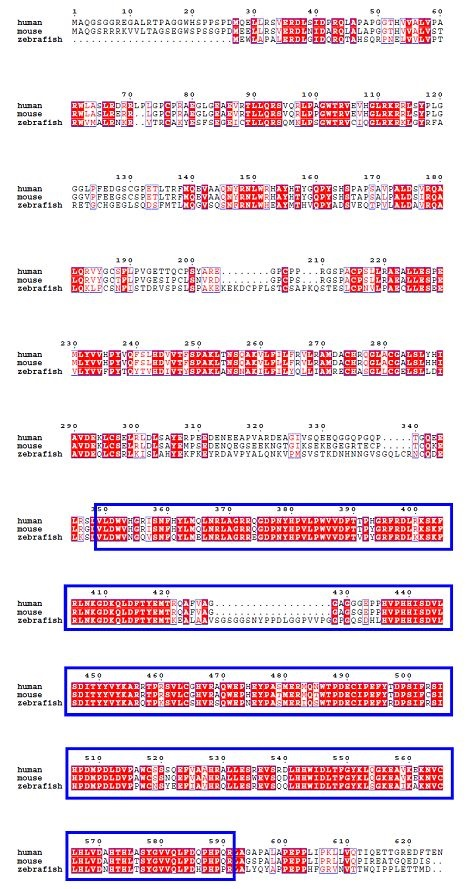


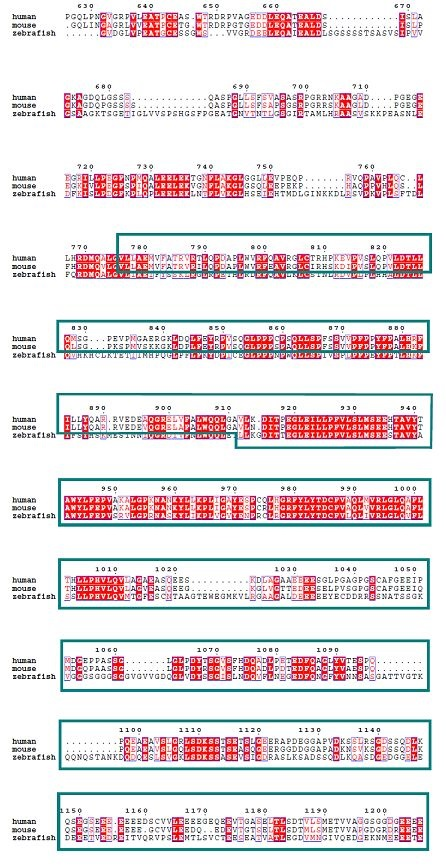


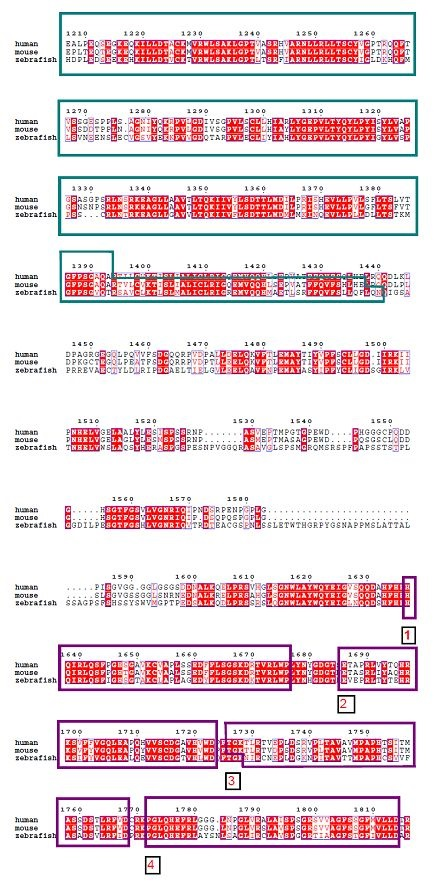


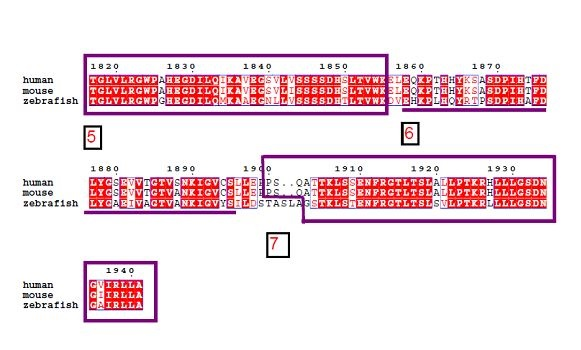

Supplement: Supplementary file 2 — 10.1186/s12868-015-0229-4 Alignment of the putative wdr81 protein in zebrafish with human WDR81 and mouse Wdr81 proteins. Alignment was analyzed with ESPript program [15]. Identical amino acid residues through the proteins of the three organisms are highlighted. The boxed areas in blue indicate BEACH domain, in green indicate MFS domain and in purple indicate WD40 repeat domains. WD40 repeat domains are marked with numbers. Number 6 is predicted to exist only in zebrafish. [file 12868_2015_229_MOESM2_ESM.doc]
