# Supplementary material for: Characterization of a novel zebrafish (Danio rerio) gene, wdr81, associated with cerebellar ataxia, mental retardation and dysequilibrium syndrome (CAMRQ)
Source: BMC Neurosci. 2015 Dec 23;16:96. doi: 10.1186/s12868-015-0229-4 (PMC4690267; doi:10.1186/s12868-015-0229-4)
Supplement: Supplementary file 3 — 10.1186/s12868-015-0229-4 Variants in 24 hpf embryo and brain wdr81 3’UTR except the 266 bp long insertion. [file 12868_2015_229_MOESM3_ESM.doc]

Additional Table S1. Variants in 24 hpf embryo and brain *wdr81* 3’UTR except the 266 bp long insertion.

| c.6506 T>C |
| --- |
| c.6707 A>T |
| c.6733 C>G |
| c.7007_7008insG |
| c.7015delT |
| c.7019_7023delACTCT |
| c.7103 T>C |
| c.7296 T>A |
| c.7331_7334delATAT |
| c.7512 A>G |
| c.7546 A>G |
| c.7770 G>T |
| c.7985 C>T |
| c.8050 A>G |
| c.8136 T>C |
| c.8252 A>G |
